# Two T7-like Bacteriophages, K5-2 and K5-4, Each Encodes Two Capsule Depolymerases: Isolation and Functional Characterization

**DOI:** 10.1038/s41598-017-04644-2

**Published:** 2017-07-04

**Authors:** Pei-Fang Hsieh, Hsiao-Hsuan Lin, Tzu-Lung Lin, Yi-Yin Chen, Jin-Town Wang

**Affiliations:** 1 Department of Microbiology, National Taiwan University College of Medicine, Taipei, Taiwan; 2 Department of Pathology and Laboratory Medicine, Taipei Veterans General Hospital, Taipei, Taiwan; 3 Department of Pediatrics, Chang Gung Children’s Hospital, Chang Gung Memorial Hospital, Chang Gung University, College of Medicine, Taoyuan, Taiwan; 4 Department of Internal Medicine, National Taiwan University Hospital, Taipei, Taiwan

## Abstract

Two *Klebsiella* bacteriophages K5-2 and K5-4, which are able to infect and grow on either capsular types K30/K69 and K5 or K8 and K5 of *Klebsiella* strains, were isolated and characterized. Each phage contained two open reading frames (ORFs), which encoded two putative capsule depolymerases, respectively. The first ORF encoded tail fiber proteins, which have K30/K69 depolymerase and K8 depolymerase activities. The second ORF encoded hypothetical proteins, which are almost identical in amino acid sequences, and have K5 depolymerase activity. Alcian blue staining of enzyme-treated capsular polysaccharides (CPS) showed that purified depolymerases can cleave purified *Klebsiella* CPS *in vitro* and liberate monosaccharaides. Capsule K5 deletion mutants were not lysed by either phage, suggesting that the capsule was essential for phage infection. Bacterial killing was observed when incubated *Klebsiella* strains with phages but not with purified depolymerases. Treatment with the K5-4 phage significantly increased the survival of mice infected with a *K*. *pneumoniae* K5 strain. In conclusion, two dual host-specific *Klebsiella* phages and their tailspikes exhibit capsule depolymerase activity were characterized. Each phage and phage-encoded depolymerase has specificity for capsular type K30/K69, K8 or K5, and could be used for the typing and treatment of *K*. *pneumoniae* infection.

## Introduction

*Klebsiella pneumoniae* is an opportunistic Gram-negative bacterium that causes several hospital-acquired infections as well as community-acquired pyogenic liver abscess (PLA) and pneumonia^1–10^. The capsule is an important virulence factor in *K*. *pneumoniae*-caused PLA and pneumonia, and capsular types are related to the clinical manifestation of the infections^11, 12^. K1, K2, K5, K20, K54 and K57 are the six predominant capsular types of PLA and pneumonia strains in Taiwan^9, 13^. Among these six capsular types, K1, K2, K5 and K57 also account for PLA in Singapore^14^. To date, most of the strains causing PLA remain susceptible to a wide range of antimicrobial drugs^15^. However, the occurrence of antibiotic-resistant PLA strains has been reported^16, 17^. Notably, the global emergence of extended spectrum beta-lactamases (ESBLs) and carbapenem resistance in *K*. *pneumoniae* is a serious problem^18^; few remaining last-resort antibiotics can be used for treatment of carbapenem-resistant *K*. *pneumoniae* (CRKP) infection. Therefore, early diagnosis and treatment for these invasive diseases are required.

To date, a total of 79 capsular types have been identified and associated with different *Klebsiella* species^19^, including 77 types from reference strains recognized by serological reactivity tests established during the period 1926 to 1977^20^ and 2 new types of *K*. *pneumoniae* (KN1 and KN2) characterized by molecular genotyping and phage typing recently^21, 22^. Polymerase chain reaction-based genotyping of the capsular polysaccharide synthesis region, *cps*-PCR genotyping, was first adopted for the detection of specific *wzy* genes in *Klebsiella* spp. type K1^23, 24^. Recently, *wzi* or *wzc* sequencing was also used for *Klebsiella* spp. capsular typing^19, 25^. Of note, there are at least >130 capsule types have been identified by analysis of capsule synthesis loci so far^26^. However, some studies documented that most of the CRKP strains from the Europe and the United States were assigned to a ST258 clone by multilocus sequence typing (MLST), several novel *cps* synthesis regions were identified but capsular types were not well defined^27–29^.

Bacterial polysaccharide capsules have evolved to protect the bacterium from the host immune system and to limit infection by some types of bacteriophages. However, capsules can also act as receptors for other types of phages that contain capsule depolymerases. Phages able to infect *Klebsiella* K5 strains and phage-borne depolymerases were isolated in 1980s, but the sequences of these phages or phage-borne depolymerases were not available^30, 31^. Phage depolymerases, which are often a part of the tail spike or tail fiber, can degrade bacterial capsular polysaccharides into their oligosaccharide units during infection. As such, the use of phages for *Klebsiella* capsular typing has been previously described^32, 33^. Such lytic phages can also be used as therapeutic agents^34–36^. In addition, phages are strain-specific, highly efficient against biofilms, and easy to isolate and manipulate. Capsule depolymerases have multiple applications, including use as therapeutic agents against bacterial pathogens^37, 38^, for the prevention or eradication of biofilms^39, 40^, and for the production of oligosaccharides from polysaccharides^39–41^. Our previous study has indicated that a bacteriophage (NTUH-K2044-K1-1) and its capsule depolymerase exhibit specificity for encapsulated *K*. *pneumoniae* K1 and can be used for the treatment of K1 *K*. *pneumoniae* infection^42^. In addition, we determined the capsular types of CRKP in Taiwan using *wzc* sequencing and confirmed that K64, which accounted for 32/85 CRKP strains (38%), was the most prevalent type. In that study, ST11 was the major ST type of these CRKP strains. Thus, we isolated a K64-specific depolymerase and utilized it for the treatment of a K64 CRKP infection in a murine model^43^.

In this study, we isolated and characterized two *Klebsiella* bacteriophages K5-2 and K5-4, that each encodes two capsule depolymerases against K30/K69 and K5 or K8 and K5. We examined the host spectra of these two phages and determined the specificities of their-borne capsule depolymerases. We also compared the sensitivities of these two phages and capsule depolymerases in *Klebsiella* typing. We further validated the capsule degrading properties of these capsule depolymerases by Alcian blue staining of enzyme-treated capsular polysaccharides. We demonstrated the phage killing effects of phages K5-2 and K5-4 in *Klebsiella* strains and investigated the clinical potential of the phage K5-4 treatment for a K5 *K*. *pneumoniae* strain in a murine model of bacteremia.

## Results

### Isolation and characterization of the *Klebsiella* bacteriophages, K5-2 and K5-4

**Figure 1:**
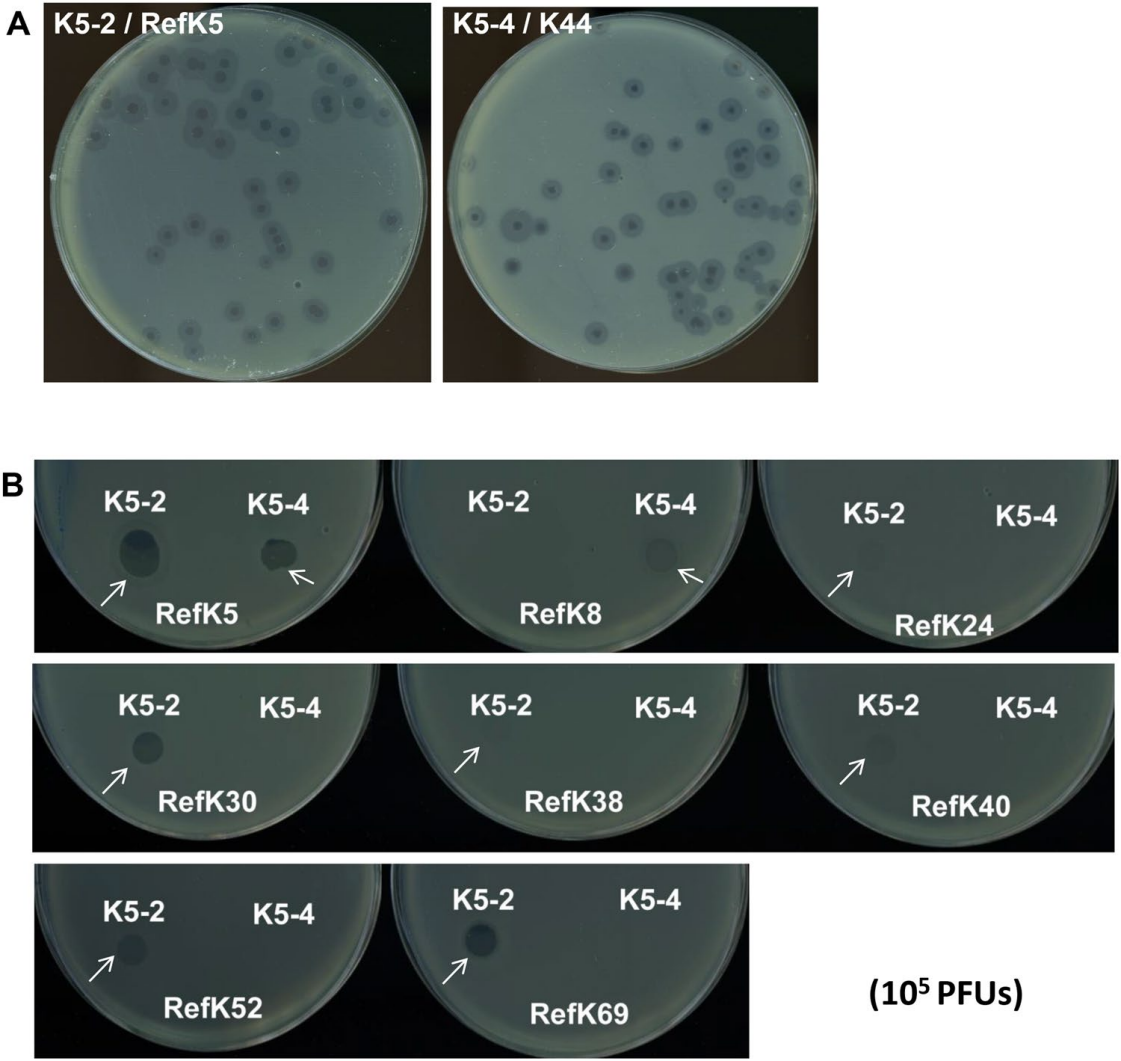
Plaque shape and spot tests of phages K5-2 and K5-4. (**A**) Clear plaques surrounded by translucent halos are observed on the plate with phages K5-2 and K5-4 filtrate incubated with the *Klebsiella* reference K5 strain and *K*. *pneumoniae* NTUH-K44 strain. (**B**) Spot tests of K5-2 and K5-4 (10^5^ PFU) on 77 *Klebsiella* K serotype reference strains and two new type N1 and N2 strains. The reference K5 strain was used as a positive control (positive reactions are indicated by a white arrow). Negative reactions are observed in the other reference strains (data not shown).

We first sought to isolate a lytic phage of the K5 *Klebsiella* strain from the natural environment. Two phages were isolated from sewage collected from Taipei by co-incubation with two capsular type K5 *Klebsiella* strains, the reference K5 and the *K*. *pneumoniae* NTUH-K44 (capsular type K5), respectively. In spot tests, the K5-2 phage caused a lytic spot on the *Klebsiella* reference K5 strain; K5-4 phage caused a lytic spot on the *K*. *pneumoniae* NTUH-K44 strain. In addition, the K5-2 phage caused lytic infections and plaque formation on the *Klebsiella* reference strain, K5; the K5-4 phage caused lytic infections and plaque formation on the NTUH-K44 strain (Fig. 1A).

### The extended host range of K5-2 and K5-4

Previous studies have indicated that the translucent halos surrounding plaques resulted from capsule-degrading enzymes from the phage^44^. Therefore, we assessed infection by both K5-2 and K5-4 phages on well-characterized 77 capsular type *Klebsiella* reference strains, as well as KN1 and KN2 capsular type reference strains^21, 22^. Spot tests revealed that the K5-2 phage not only infected K5 strains, but also caused spots on the *Klebsiella* reference strains with different capsular types, including K5, K24, K30, K38, K40, K52 and K69. The K5-4 phage also caused spots on the K5 and K8 *Klebsiella* reference strains (Fig. 1B). Plaque assays revealed that the K5-2 phage caused lytic infections and plaque formation on the reference *Klebsiella* strains with capsular types K5, K30 and K69, whereas the K5-4 phage caused lytic infections and plaque formation on the reference *Klebsiella* strains with capsular types K5 and K8 (Table 1).

**Table 1:** Host range of phages K5-2 and K5-4.

Phage	Spotting host range	Plaguing host range
K5-2	K5 K24 K30 K38 K40 K52 K69	K5 K30 K69
K5-4	K5 K8	K5 K8

### Full genome sequences of phages K5-2 and K5-4

We further characterized the genome of the K5-2 and K5-4 phages using high-throughput sequencing. K5-2 has a circular map of the 41,116 bp genome, which has a G + C content of 47.5% and 42 predicted ORFs. K5-4 has a circular map of the 40,163 bp genome, which has a G + C content of 45.4% and 42 predicted ORFs. BlastN analysis revealed that the most related genome sequences in the database were *Klebsiella* phage K11 (accession EU734173.1, 86% query coverage and 94% identity), *Klebsiella* phage vB_KpnP_KpV763 (accession KX591654.1, 86% query coverage and 93–94% identity), *Klebsiella* phage vB_Kp1 (accession KT367885.1, 86–87% query coverage and 93% identity) and *Enterobacteria* phage K30 (accession HM480846.1, 85–86% query coverage and 91–92% identity). In addition to sequence similarity, they exhibited synteny with these related phages (Figure S1). The results of genome structure and sequence similarity all suggested classification of these phages are Podoviridae members and belong to T7-like viruses. With the exception of the tail fibers, *Klebsiella* phages K11, vB_KpnP_KpV763, vB_Kp1 and *Enterobacteria* phage K30 have structural proteins which are arranged similar on the genome and with high sequence identity with the corresponding proteins of phages K5-2 and K5-4. The full genome sequences of phages K5-2 and K5-4 showed high similarity (90% coverage and 93% identity; blastn alignment) and were flanked with 177 or 179 bp direct-repeat sequences at the 2 ends (Figure S2A). The upstream gene encodes internal virion protein D (gp16), which have 94% nucleotide sequence identity. The downstream gene encodes gp17.5 protein and sharing a high nucleotide sequence similarity of up to 99%. Therefore, we developed a PCR method for determination of two putative capsule depolymerases in T7-like phages using the conserved primer pairs, enz1-F and enz1-R, which were based on the upstream and downstream genes of the tail fiber region (Figure S2B).

### The K5-2 ORF37 mutant was defective in growth on both K30 and K69 *Klebsiella*

The phage K5-2 forms clear plaques on bacterial lawns of K30, K69 and K5 *Klebsiella* strains; therefore, we hypothesized that mutants in either tail spike would result in no plaque formation due to growth of the nonpermissive host. Using a selection/amplification method to enrich for those phages that can replicate on K5 but not K69 hosts^45^, one isolate, K5-2 ORF37 mutant, was further characterized and found to be unable to grow on any of the K30 or K69 strains (Figure S3). The infectivity of phage K5-2 to *Klebsiella* strains with capsular types K5, K30 and K69 strains (including the reference strains and 6 clinical strains) was observed. In contrast, the K5-2 ORF37-deletion mutant phage was able to infect K5 strains, but lost infectivity to K30 and K69 strains. Besides the gene deletions between the nucleotide 448 of *orf37* to nucleotide 26 of *orf38*, the K5-2 ORF37 mutant contains all the intact ORFs of its parental strain, K5-2. The K5-2 ORF37 mutant carried a fused gene in *orf38* and retained its infectivity of K5 strains, implying that *orf38* might encode the K5 depolymerase.

### Identification of K5-2 ORF37, K5-2 ORF38 and K5-4 ORF38 as putative capsule depolymerases

We observed that the plaques of the K5-2 phage were surrounded by halos in the K5, K30, and K69 strains, indicative of bacterial cell decapsulation, whereas the plaques of the K5-4 phage were surrounded by halos in the K5 and K8 strains. This observation suggested that these phages produced depolymerase enzymes that could diffuse through the agar layer^46^. Analysis of the genome sequences revealed that *orf37* (2379 bp) of phage K5-2 encoded a putative protein of 792 amino acids and *orf37* (2250 bp) of phage K5-4 encoded a putative protein of 749 amino acids, which have 35% sequence identity. The down-stream genes, *orf38* of phage K5-2 and *orf38* of phage K5-4, encoded a hypothetic protein (685 and 684 amino acids), respectively, sharing amino-acid sequence identity of up to 98%.

**Figure 2:**
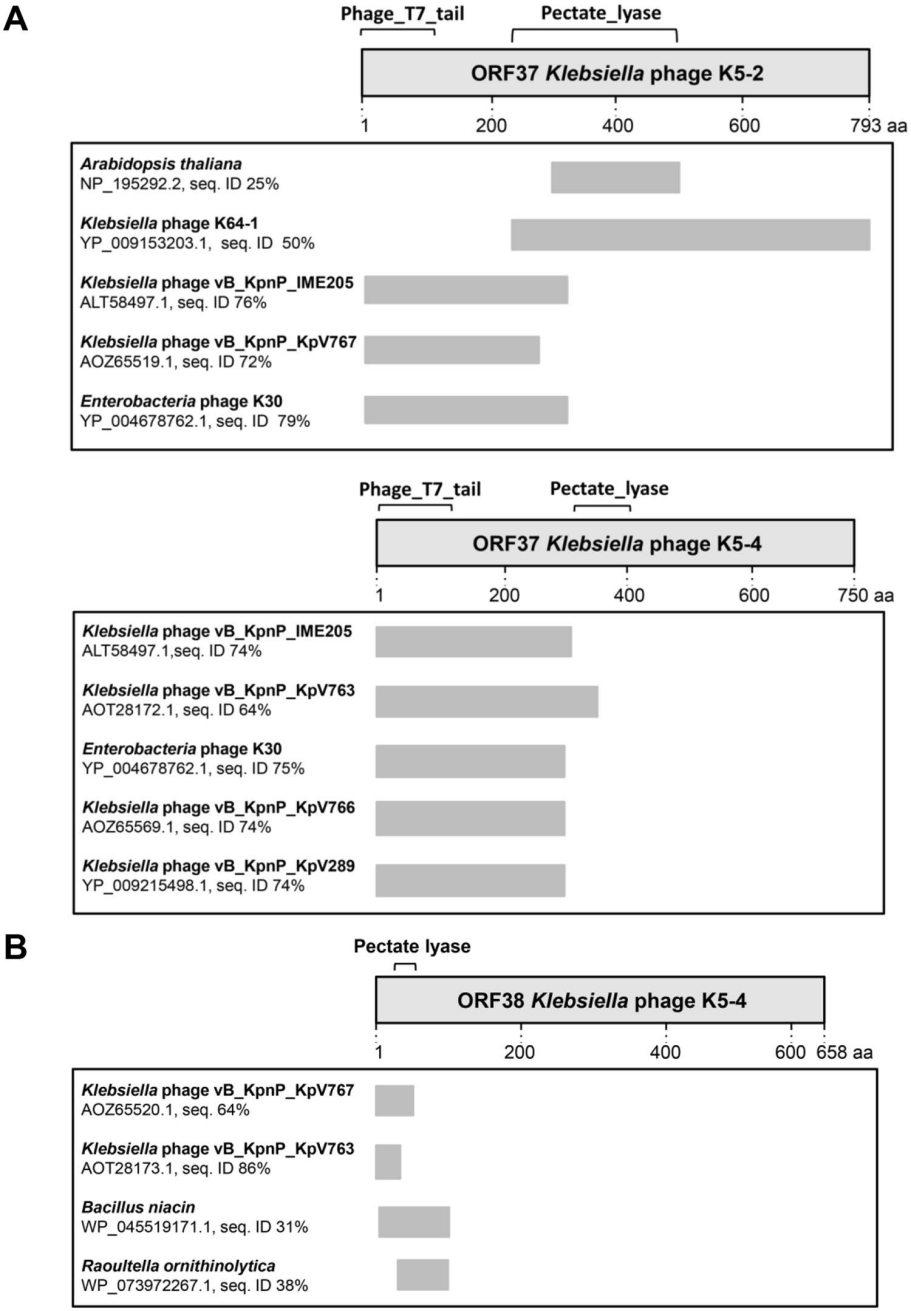
Bioinformatic analysis of *Klebsiella* phages K5-2 and K5-4. (**A**) NCBI SmartBLAST analysis of K5-2 ORF37 and K5-4 ORF37 proteins in non-redundant sequence database. (**B**) NCBI SmartBLAST analysis of K5-4 ORF38 protein in non-redundant sequence database.

NCBI SmartBLAST analysis of the K5-2 *orf37* and K5-4 orf37 gene products (putative tail fiber proteins) showed moderate sequence identity of the N-terminal region to other known phage tail fiber proteins of *Klebsiella* phage vB_KpnP_IME205, *Klebsiella* phage vB_KpnP_KpV767, *Enterobacteria* phage K30, *Klebsiella* phage vB_KpnP_KpV763, *Klebsiella* phage vB_KpnP_KpV766 and *Klebsiella* phage vB_KpnP_KpV289 (Fig. 2A). All of these tail fiber proteins contain conserve domain (residues 1–157) of the phage T7 tail fiber protein in the N-terminus. Amino acid sequences of these two ORF37 proteins also evidenced a fragment exhibiting similarity to the pectate lyase-like superfamily protein (residues 295–351). Moreover, the C-terminal region of the K5-2 ORF37 has significant sequence identity (50%) to a putative structural protein, a recently identified *Klebsiella* K30/K69 depolymerase from the *Klebsiella* Phage K64-1 (ORF S2-6)^47^. A comparison of K30/K69dep of phage K5-2 with ORF S2-6 protein exhibited only limited amino acid sequence identity (792/767 [37%]) across the entire lengths of the two proteins, indicating that there are variant depolymerases specific for the same capsule type of *Klebsiella*. In the case of the K5-4 *orf38* gene product, four moderate homologs of the N-terminal region were identified (Fig. 2B). Those included a hypothetical protein of *Klebsiella* phage vB_KpnP_KpV767, a putative tail fiber protein of *Klebsiella* phage vB_KpnP_KpV763 and hypothetical proteins of *Bacillus niacin* and *Raoultella ornithinolytica*. Consistently, ORF38 of phage K5-4 possesses a pectate lyase domain (residues 37–166) in the N-terminal region. Notably, none of the aforementioned phage proteins has been characterized *in vitro*.

### Expression of capsule depolymerases specific for K5, K8, and K30/K69

**Figure 3:**
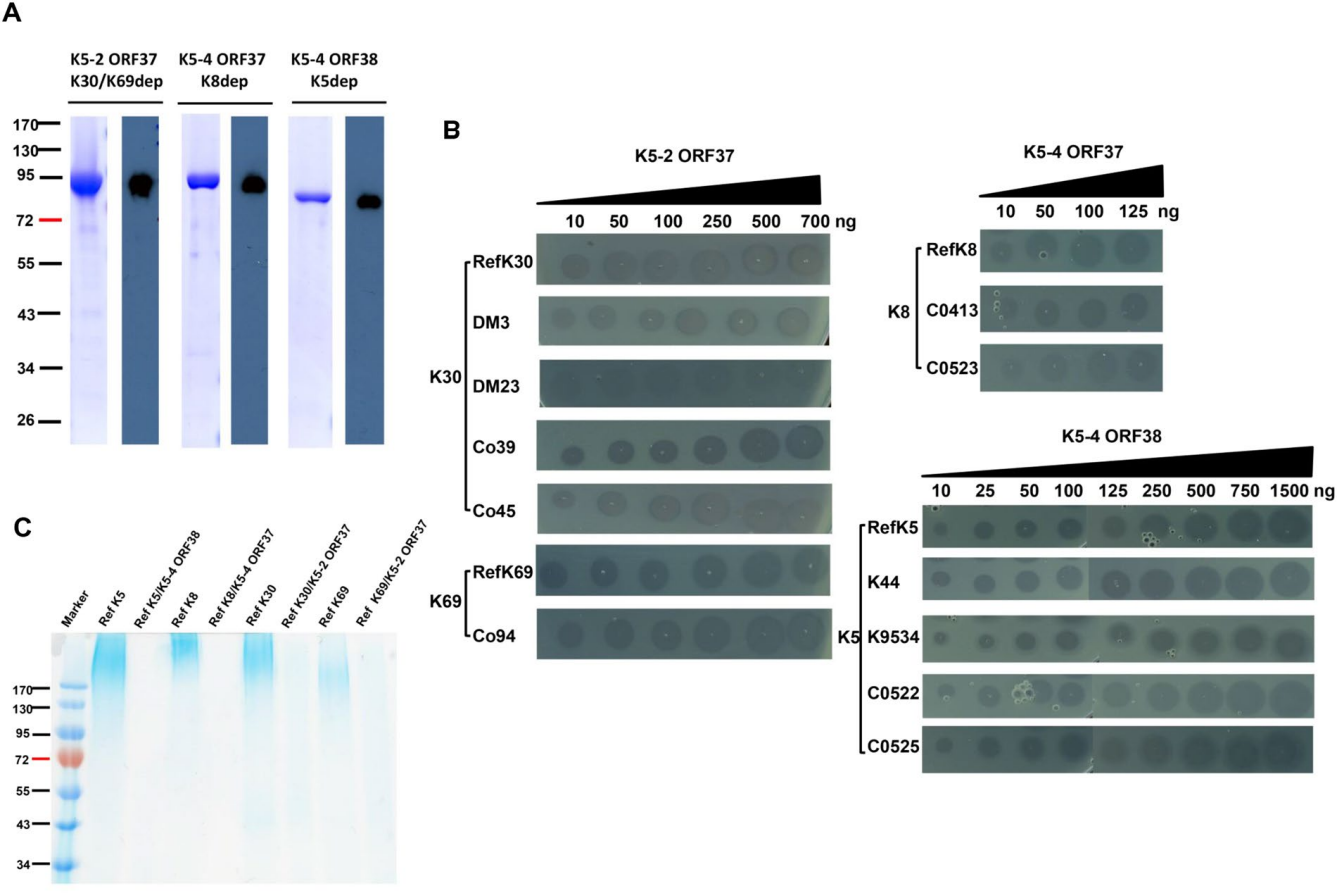
Capsule depolymerase expression of phages K5-2 and K5-4 and enzymatic activity of their purified capsule depolymerases. (**A**) Purity of recombinant K5-2 ORF37, K5-4 ORF37 and K5-4 ORF38 proteins. The purified K5-2 ORF37, K5-4 ORF37 and K5-4 ORF38 proteins were separated on SDS-PAGE with Coomassie blue staining, and the molecular weights were indicated beside the protein markers. Immunoblots showing the purified recombinant proteins were separated by SDS-PAGE, transferred onto a nitrocellulose membrane, and detected using a mouse anti-His antibody (1:5000) and a rabbit anti-mouse IgG-HRP (1:10,000). (**B**) Activity of purified K5-2 ORF37, K5-4 ORF37 and K5-4 ORF38 proteins among different capsular types in K30, K69, K8 and K5 *Klebsiella* strains. Five K30 strains (ref K30, DM3, DM23, Co39 and Co45 and two K69 strains (ref K69 and Co94) were spotted with K5-2 ORF37 protein (10–700 ng). Three K8 strains (ref K8, C0413 and C0523) and five K5 strains (ref K5, K44, K9534, C0522 and C0525) were spotted with K5-4 ORF37 protein (10–125 ng) and K5-4 ORF38 protein (10–1500 ng). (**C**) Alcian blue staining of CPS treated with K5, K8 and K30/K69 capsule depolymerases. Polysaccharide extracts from the reference K5, K8, K30 and K69 strains were treated with 5 μg K5dep, K8dep and K30/K69dep, respectively, and stained with Alcian blue.

To determine whether the product of these genes had capsule depolymerase activity, we cloned *orf37* of K5-2, *orf37* of K5-4, and *orf38* of K5-4 into the pET28c expression vector, respectively. His-tagged proteins were expressed and purified, here designated as K30/K69dep, K8dep and K5dep (Fig. 3A). In spot tests, the purified ORF37 protein of K5-2 caused decapsulation in both K30 and K69 strains (including the K30, K69 reference strain and their 5 clinical strains) but not in the 77 non-K30 and non-K69 reference strains. The purified ORF37 and ORF38 proteins of K5-4 caused decapsulation in K8 (including the K8 reference strain and 2 clinical strains) and K5 strains (including the K5 reference strain and 4 clinical strains) but not in the 78 non-K8 or non-K5 reference strains, respectively (Fig. 3B). The capsule degrading properties of K5dep, K8dep and K30/K69dep were further validated by Alcian blue staining of enzyme-treated CPS, respectively. Our results revealed that enzyme-free CPS showed high-molecular weight capsular polysaccharide polymers at the top of an SDS-PAGE gel, whereas lower-molecular weight material (that was weakly detected) was observed when CPS was treated with 5 μg of enzyme (Fig. 3C). Thus, each phage contained two ORFs, the first encodes K30/K69 depolymerase or K8 depolymerase and the second encodes K5 depolymerase.

### Application of the capsule depolymerase for capsular typing

The sensitivity for capsular types of both phages was evaluated among four capsular types, K30, K69, K8 and K5, in *K*. *pneumoniae* strains, whose capsular type was determined by PCR using wzc primers^25^. As expected, the K5-2 phage could infect all tested strains with capsular types K30 and K69 (including the K30, K69 reference strain and their 5 clinical strains), whereas K5-4 could infect all tested strains with capsular types K8 and K5 (including the K8, K5 reference strain and their 6 clinical strains) (Figure S4A). However, the infectivity of both K5-2 and K5-4 phages was different (by up to 10^4^-fold) among the same capsular type strains (Figure S4B). The infectivity of phage K5-2 to the *Klebsiella* reference K5 strain and PLA-associated K9534 strains was significantly higher than that to another PLA-associated K44 strain. Similar results with capsular type K30 strains were obtained. The infectivity of phage K5-4 to the PLA-associated strains, K9534 and K44, was significantly higher than that observed with the reference *Klebsiella* K5 strain (*P* < 0.001; generalized linear model and generalized estimating equations [GLM/GEE] test).

In contrast to phage infection, spotting with aliquots of ≥10 ng of the purified recombinant depolymerases, ORF37 of K5-2, ORF37 of K5-4, and ORF38 of K5-4, consistently produced a translucent spot for each of their host strains (Fig. 3B). After validation of the sensitivity and specificity in more strains (including the K30, K69 reference strains and their 5 clinical strains; the K8, K5 reference strains and their 6 clinical strains), the purified depolymerase exhibited a consistent sensitivity between different strains with the same capsular type, suggesting that capsule depolymerase may provide a more consistent result in capsular typing than exposure to the whole phage.

### Each K5-2 virion contains both tail proteins

**Figure 4:**
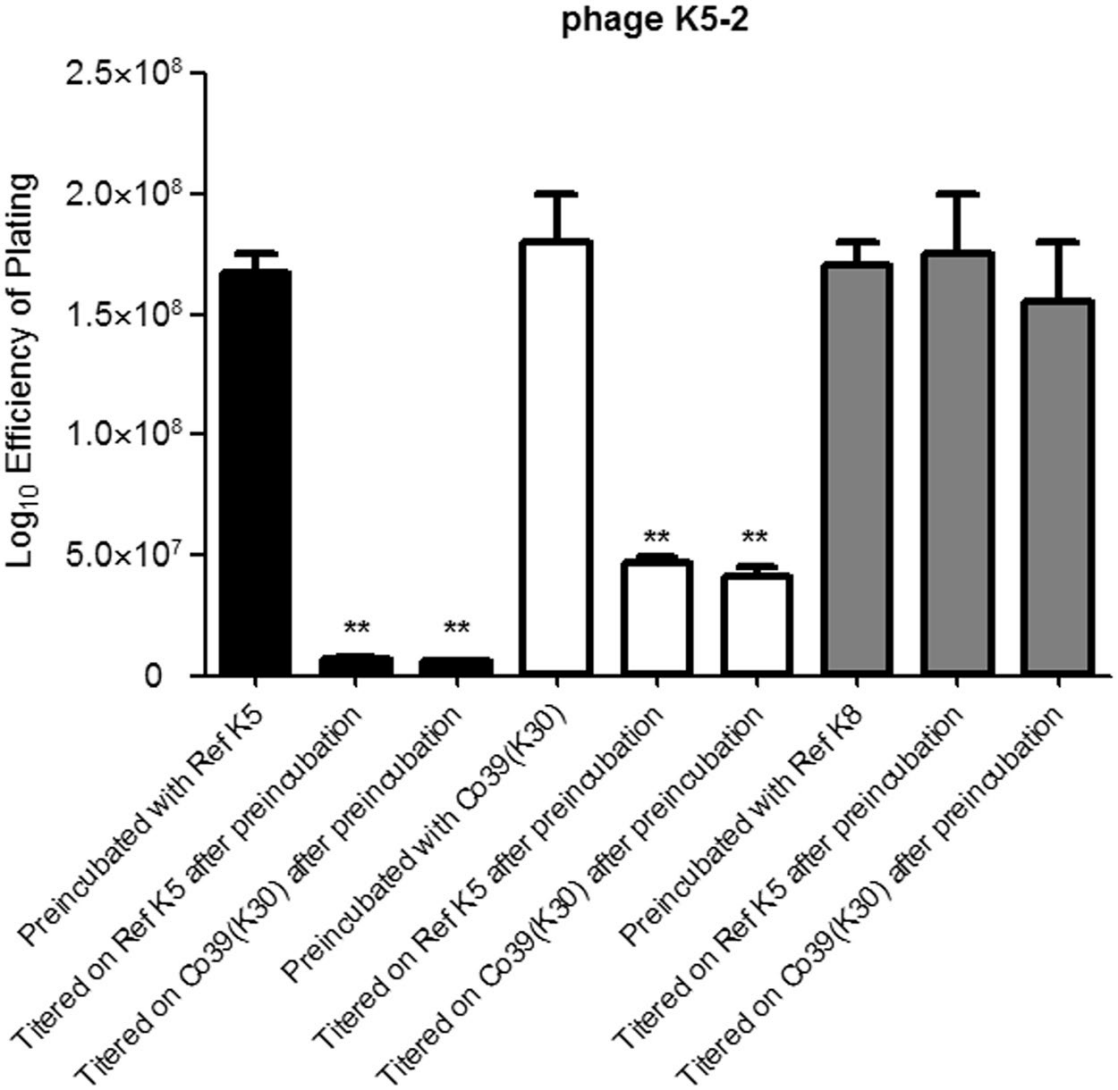
Adsorption experiments of phage K5-2. Preincubation of phage K5-2 with its host the K5 reference or Co39 (K30) strain and determination the viral titers on the reference K5 and Co39 (K30) strains, respectively. Negative reactions were observed on the reference K8 strain. The data represent the means of three independent trials; the error bars represent the standard deviations. **P < 0.01 by Student’s *t* test (compared to the viral titer of the respective host before incubation).

We next addressed the question of whether K5-2 particles contain both tail fiber proteins, or if two populations of particles (one containing the K30/K69 depolymerase and the other containing the K5 depolymerase) were produced after infection. We made a phage adsorption experiment according to the method from one previous study^48^ and using the reference K5 strain as a host and determined its titer on reference K5 and Co39 (K30) strains (Fig. 4). If each of the phage particles contained both tail proteins, titers of the phage remaining in the filtrate would be the same on the two strains. After incubation with the reference K5 strain, phage K5-2 titer was reduced by titering on reference K5 or Co39 (K30) strain. Similar results were seen in the converse experiment in which the 5-min incubation was performed with the Co39 (K30) strain. As controls, we performed similar experiments using the reference K8 strain for the incubation. In this case, phage K5-2 titer was not reduced by titering on reference K5 or Co39 (K30) strain. We found that each virion has both the K5 and K30/K69 depolymerases.

### Infectivity of the phage K5-2 and K5-4 to a capsule K5 deletion mutant

**Figure 5:**
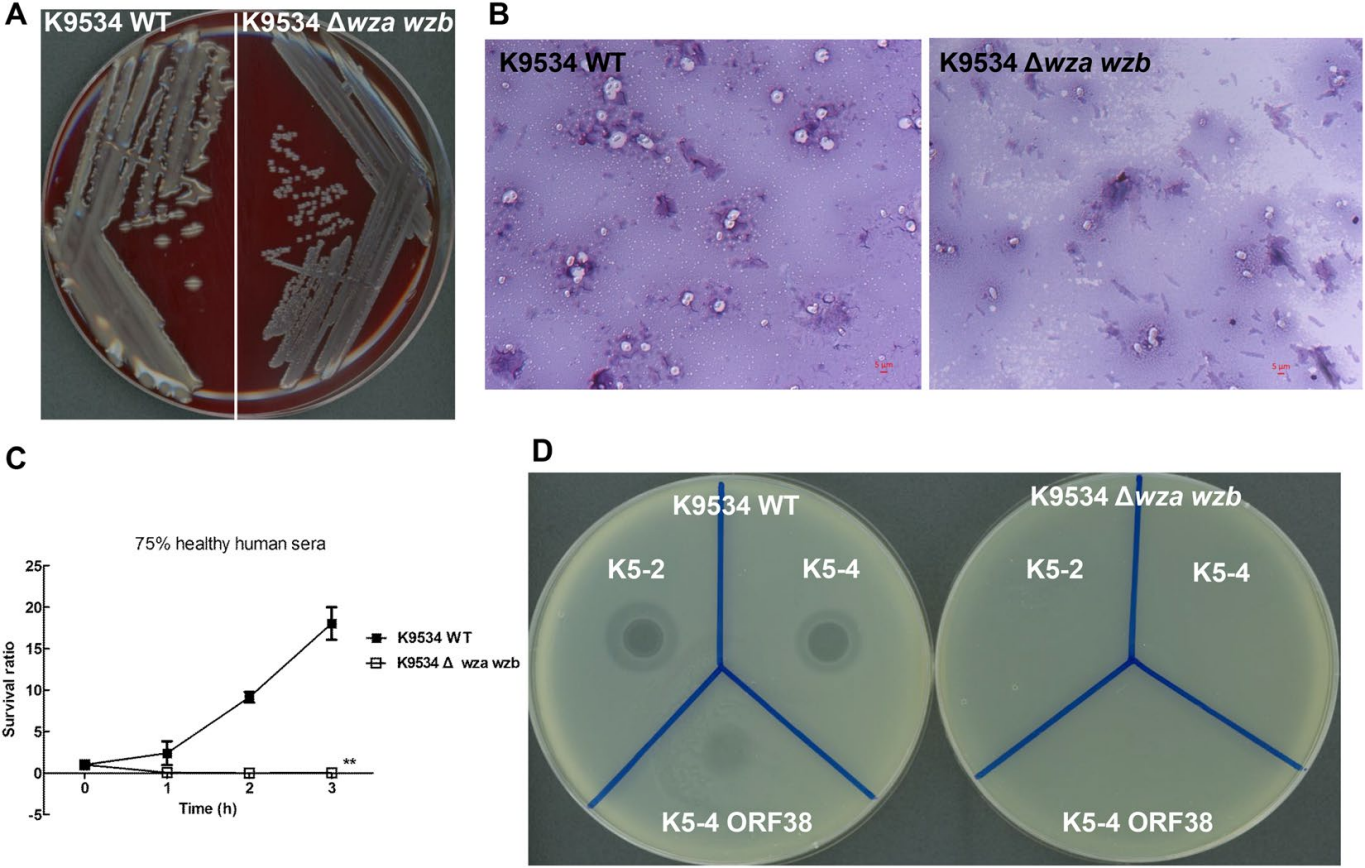
Characteristics of the NTUH-K9534 wild-type and its isogenic capsule deficient strains. (**A**) Differences in the hypermucoid phenotype are observed between the wild-type and its capsule deficient strains on the blood agar. (**B**) Under light microscopy, the wild-type strain K9534 produces an exopolysaccharide web attached to the capsule (original magnification, 1,000). The K9534 *wza wzb*-deletion mutant loses the capability of producing an exopolysaccharide capsule. (**C**) Serum sensitivity assays of resistance to killing by nonimmune healthy human serum of the K9534 wild-type and its *wza wzb*-deletion mutant strains. The data represent the means of three independent trials; the error bars represent the standard deviations. A mean survival ratio ≥1 corresponds to serum resistance. **P < 0.01 by Student’s *t* test (compared to the wild-type strain). (**D**) Spot tests of phages K5-2, K5-4 and the K5-4 ORF38 protein on the K9534 wild-type and its capsule deletion mutant strains. Phages (10^6^ PFUs) or 1 μg K5 depolymerase were spotted on the plate, and a clear spot or a translucent spot, respectively, is observed.

Because phages K5-2 and K5-4 selectively infected strains with the same capsular types, we further examined whether the bacterial capsule was essential for infection by the phages. We generated an isogenic K9534 capsule-deficient mutant, in which *wza* and *wzb* (predicted to encode Wza and Wzb) of the *cps* loci were deleted by an unmarked deletion method using the temperature-sensitive plasmid pKO3-Km^49^. Differences in the hypermucoid phenotype (formation of viscous strings ≥5 mm in length represents a positive string test)^12^ were observed between the wild-type and its capsule deficient strains (Fig. 5A). The K9534 wild-type showed mucoid colony appearance on the blood agar plate and presented a positive string test. In contrast, the K9534 *wza wzb*-deletion mutant exhibited nonmucoid colony appearance and presented a negative string test. Under light microscopy with Congo red stain, the K9534 wild-type produced a mucoviscous exopolysaccharide capsule, whereas its isogenic *wza wzb*-deletion mutant did not produce the exopolysaccharide capsule (Fig. 5B). Additionally, this capsule deficient mutant also exhibited a marked decrease in serum resistance compared with the wild-type. The K9534 *wza wzb*-deletion mutant was extremely sensitive to nonimmune human serum, which killed all of the mutants in 1 hour (Fig. 5C).

We repeated the spot tests and compared the infectivities of the K9534 wild-type and its capsule-deficient mutant by phages K5-2 and K5-4. A clear spot surrounded by a translucent halo and a translucent spot are observed when phages K5-2, K5-4 and the K5-4 ORF38 protein were spotted on the plate overlaid with top agar containing the wild-type strain, respectively (Fig. 5D). In contrast to the parental strain, the *wza wzb*-deletion mutant was not infected by either phage. Furthermore, the purified ORF38 protein of the K5-4 phage caused decapsulation in the wild-type but not in the *wza wzb-*deletion mutant. Therefore, this depolymerase was specific for K5 capsular polysaccharides, suggesting that infection of the phage might occur via targeting and recognition of the capsular polysaccharides in *K*. *pneumoniae* such that they might be the receptor required for phage infection.

### Effects of phage or capsule depolymerase treatment in *Klebsiella*

**Figure 6:**
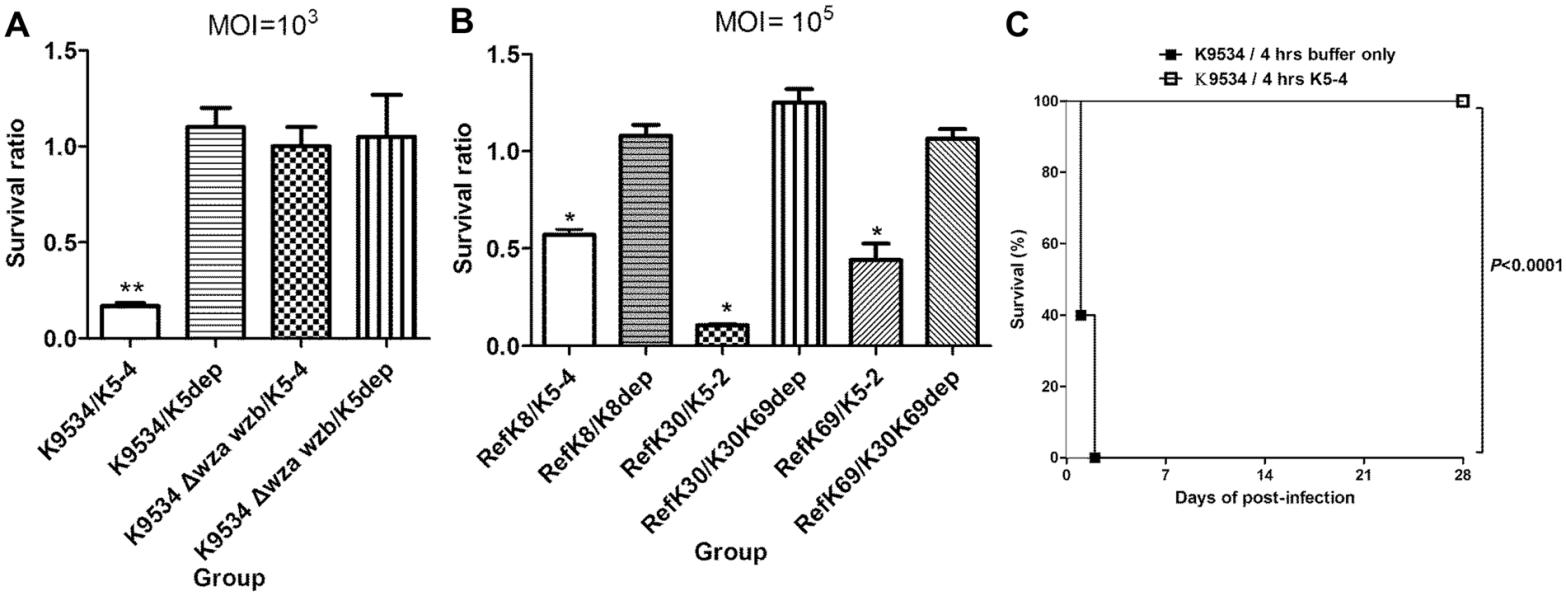
Killing effects of phage treatment. (**A**) Killing effects of phage treatment and capsule depolymerase treatment on the *Klebsiella* strains. The K9534 and its capsule deletion mutant strains were incubated with the K5-4 phage (multiplicity of infection, 1000) and 10 μg K5 depolymerase. The survival bacterial counts after 30 minutes compared with initial counts were represented by the survival ratio from 3 independent experiments (mean ± standard deviation [SD]). **P < 0.01 by Student’s *t* test (compared to enzyme-treated group). (**B**) The *Klebsiella* reference K8, K30 and K69 strains were incubated with the K5-4 phage, K5-2 phage (multiplicity of infection, 1000) and 10 μg CPS depolymerase, respectively. The survival bacterial counts after 30 minutes compared with initial counts were represented by the survival ratio from 3 independent experiments (mean ± standard deviation [SD]). ^*^ *P* < 0.05 by Student’s *t* test (compared to enzyme-treated group). (**C**) Treatment efficacy of the phage K5-4 in mice. Groups of ten infected mice received either no phage or a single dose of the K5-4 phage administered intraperitoneally 4 h after the *K*. *pneumoniae* NTUH-K9534 infection. Treatment with the K5-4 phage (*P* < 0.0001; log–rank test) significantly increased the survival of mice infected with the NTUH-K9534.

The effects of treatment with phage K5-4 and K5dep protein in the K9534 wild-type and its capsule deficient strains were compared *in vitro*. After treatment with phage K5-4 (multiplicity of infection, 1000) for 30 minutes, 90% of the K9534 wild-type bacteria were killed. In contrast, the killing effect of this phage in the K9534 *wza wzb*-deletion mutant was not observed (Fig. 6A). Treatment with 10 μg of the K5dep protein did not kill the K9534 wild-type and its capsule deficient strains. Similar results were obtained after treatment with the respective phage at a higher MOI (10^5^) in the reference *Klebsiella* K8, K30 and K69 strains (Fig. 6B). Thus, phage-treated bacteria significantly reduced the bacterial survival in comparison with enzyme-treated bacteria.

### Treatment with phage K5-4 in mice infected with a K5 *K. pneumoniae* strain

Because we demonstrated that the phage K5-4 killed bacteria by bacterial lysis, we then evaluated the treatment efficacy of this phage in mice infected with a K5 *K*. *pneumoniae* strain. An *in vivo* model of *K*. *pneumoniae* infection was established in mice using the wild-type NTUH-K9534 with LD_50_ values of 5 × 10^5^ CFU. IP infection with 1.7 × 10^6^ CFU of NTUH-K9534 caused death in 100% (10/10) of mice within 2 days of post-infection. In contrast, no deaths were observed among IP infected mice treated with one dose of K5-4 phage (1.5 × 10^9^ PFU) at 4 h after infection (Fig. 6C). Thus, treatment with this phage significantly increased the survival of mice infected with the PLA K5 strain, NTUH-K9534 (*P* < 0.0001).

## Discussion

We isolated two *Klebsiella* bacteriophages, K5-2 and K5-4, which are able to infect and grow on capsular types K30, K69 and K5 or K8 and K5 strains. It appears that the ability of each phage to replicate on these strains is due to the fact that each encodes two different capsule depolymerases. One is a putative tail fiber protein that allows the phage to attach and degrade either the K30/K69 or K8 capsular polysaccharide. The other is almost identical in its amino acid sequence that allows both phages to attach and degrade the K5 capsular polysaccharide. This is the first example of a *Klebsiella* phage that has dual host specificity based on having two different capsule depolymerases.

Excluding the tail proteins-encoding region, we found that all six T7-like phages exhibit genome sequences similarity, suggesting that these phages are all closely related and differ mainly in their tail fiber proteins. It, therefore, appears that there is a family of dual host-specific phages carrying two different capsule-degrading enzymes and these genes are arranged in a modular or cassette configuration. It is worth noting that the corresponding regions of the other T7-like phages might also contain two different capsule-degrading enzymes, which shared very limited sequence identity. Therefore, whether these genes encode the tail fiber proteins or structural proteins possess depolymerization activities toward other capsular types await further analyses.

In this study, we used two different techniques, spot testing and plaquing, to identify the phages and measure their host ranges. In comparison with phage plaquing, spot testing can be employed as a simple and rapid initial screen to determine a phage’s bactericidal host range even in the absence of successful phage infection^50^. Spot tests of this study revealed that phage K5-2 causes spots on seven capsular types of *Klebsiella* strains, including K5, K24, K30, K38, K40, K52 and K69. By plaque assays, this phage causes lytic infection and plaque formation on the *Klebsiella* reference strains with capsular types K5, K30 and K69. In contrast, this phage only causes spots but no plaque formation on the *Klebsiella* reference K24, K38, K40 and K52 strains, indicating this phage cannot infect these four capsular types of *Klebsiella* strains successfully for production of progeny and kill the host.

Phages that are specific for capsule antigens are believed to have hydrolytic activity associated with the tail proteins, which are part of the mature virion. In the case of K5-2, we have experimental evidence that both of the depolymerases are part of each phage particle, and we have shown that the ability to grow on either of the host types can be deleted by chemical mutagenesis. In spite of the importance of the capsule in phage infection, phage infectivity varied among the *K*. *pneumoniae* strains with the same type capsule as we observed previously^42^. Differential infectivities of the phage may result from the same capsule type of *K*. *pneumoniae* strains can be found in many unrelated clones or ancestral lineages – which differ in terms of gene content^51–53^.

The structures of the *Klebsiella* K30 and K69 capsular polysaccharides differ only in the location of the pyruvic acetal, which is linked 3,4 to Gal in K30 and 4,6 to Gal in K69^54^. Earlier studies have shown that a *Klebsiella* K69 bacteriophage contains an endo-mannosidase which not only catalyzes hydrolysis of the K69 polysaccharide but also caused lysis of lawns of *Klebsiella* K30 bacteria and has been used to depolymerize the K30 polysaccharide^55^. A recently study also indicated that the deletion of ORF S2-6 (K30/K69 depolymerase) results in the inability of the *Klebsiella* phage K64-1 to infect both K30 and K69 type strains^47^. Consistent with this finding, we found that the phage K5-2 encodes a tail fiber protein with the K30/K69 depolymerase activity that depolymerize both K30 and K69 polysaccharides. Thus, these results show that the position of the pyruvic acetal on the repeating unit of K30 and K69 capsules is unimportant for phage-borne enzyme binding and lysis.

BLAST results indicated that K30/K69 depolymerase of K5-2 and K8 depolymerase of K5-4 contain T7-phage tail domain in the N-terminal region, as well as to other known tail fiber proteins of T7-like phages. K30/K69 depolymerase, K8 depolymerase and K5 depolymerase all exhibited limited amino acid sequence similarity to other known phage proteins, indicating that the capsule type-specific depolymerase could be unique. Each of these depolymerases possesses a pectate lyase domain, indicating capsular polysaccharides might be cleaved with lyases and lead to oligosaccharides. However, the properties of these depolymerases remain unclear and await further analyses, e.g., structure, enzymatic reactions and final degradation products.

Strains with the K1 and K2 capsular types have been identified as the predominant virulent types and are prevalent in *K*. *pneumoniae* PLA^4, 10^. Unlike the virulence of the K1 and K2 strains, the NTUH-K9534 (K5) strain was less virulent (e.g., the LD_50_ of NTUH-K2044 for mice was 1 × 10^2^ CFU^12^) and exhibited very high lethal doses (LD_50_ of 5 × 10^5^ CFU). Because K5 strains were less virulent than either K1 or K2 strains, a question was raised regarding whether the K5 capsular type was more prevalent in diabetic than non-diabetic patients causing PLA. A retrospective survey recently reported that six PLA-related capsular types (K1, K2, K5, K20, K54, and K57) were more prevalent in non-diabetic patients^56^. One of our latest study indicated that K64 strains were less virulent than either K1 or K2 strains causing PLA and used high lethal doses (i.e., up to 6 × 10^6^ CFU) to challenge in cyclophosphamide-treated mice, which mimic immunocompromised mice, to monitor the therapeutic effect of capsule K64 depolymerase^43^. Therefore, these results indicate that although the PLA-associated K5 strains are less virulent than either the PLA-associated K1 or K2 strains, they are more virulent than the predominant K64 strains of CR-KP from Taiwan.

Depolymerases digest the bacterial capsule only; host innate immunity is still required for bacterial clearance. Phages can kill and amplify after targeting to the bacteria, and seem to be a better alternative treatment in immunocompromised hosts^57^. Moreover, phage with a long-term residence in tissues would improve the treatment efficacy^42, 58^. Several studies have shown that immediate bacteriophage treatment was effective in murine models of *K*. *pneumoniae* burn wound, lobar pneumonia, and K2 PLA infections^59–62^. Consistent with previous findings, the therapeutic efficacy of the *Klebsiella* phage K5-4 in a murine bacteremia model was demonstrated in this study. Therefore, phages and phage-borne depolymerases represent potential alternatives for the treatment of bacterial infections, especially in patients in whom antibiotic therapy has failed.

In conclusion, we report the isolation of two dual host-specific *Klebsiella* phages, K5-2 and K5-4, both with tail fiber proteins that exhibit capsule depolymerase activity for capsular types K30/K69 and K5 or K8 and K5. Both phages and their capsule depolymerases are expected to have clinical applications for the detection and treatment of *K*. *pneumoniae* infections.

## Materials and Methods

### Bacterial strains and culture conditions

A total of 77 K-serotype *Klebsiella* spp. reference strains were obtained from the Statens Serum Institute (Copenhagen, Denmark). Two additional strains with novel type KN1 and KN2 capsules identified in our laboratory were also included^21, 22^. Two capsular type K5 PLA isolates (NTUH-K44 and NTUH-K9534) of *K*. *pneumoniae* obtained from 1997 to 2005 in the National Taiwan University Hospital (NTUH) as described previously were used in this study^63^. Stool specimens were collected from healthy volunteers who had health checkups in the Health Management Center of the NTUH during May to November 2006, as described previously^64^. This study protocol was approved by the Institutional Review Board of National Taiwan University Hospital (IRB approval number: 9561701018). The methods were carried out in accordance with the approved guidelines and written informed consent was obtained from each participant. *K*. *pneumoniae* and *E*. *coli* strains were cultured in Luria-Bertani (LB) medium or LB medium supplemented with appropriate antibiotics, including 100 µg/mL ampicillin and 50 µg/mL kanamycin.

### Isolation of *Klebsiella* K5-2 and K5-4 bacteriophages

The *Klebsiella* reference K5 (E 5051) and the *K*. *pneumoniae* NTUH-K44 (capsular type K5) strains, were individually co-incubated with water from rivers of Taiwan in LB broth overnight. After centrifugation, the supernatant was filtered using a 0.45-µm filter and spotted onto LB plates overlaid with the respective strain to detect phage plaques. An agar overlay method was used for isolation of a pure phage preparation and to determine phage titers as described previously^33^. Single plaque isolation, elution, and re-plating were performed repeatedly.

### Determination of the host ranges of the phage and capsule depolymerase

The host spectra of the phage were determined using a spot test to observe phage infection or polysaccharide depolymerase activity^42^. Dilutions of phages were spotted on LB agar plates overlaid with top agar containing each of the different *K*. *pneumoniae* strains (Table S3). In brief, LB agar was overlaid with a top agar that had been inoculated with 200 μL of a fresh bacterial culture. Phage (1 × 10^5^ plaque forming units, PFUs) or purified recombinant polysaccharide depolymerase (1 μL, 1 μg/μL) was spotted onto the plate after the top agar had solidified. After overnight incubation at 37 °C, plates were observed for formation of lytic or semi-clear spots. An efficiency of plating assay was also used to quantify the ability of the phage to infect other strains, as described previously^33^. Briefly, samples of serial 10- fold dilutions (in SM buffer) of the respective phage suspensions were incubated for 15 min with the isolating host and with a heterologous host. Each mixture was diluted into 4 ml of LB-soft agar and layered on to an LB agar plate. The plates were incubated overnight at 37 °C and scored for the presence of plaques. The lytic activity of phage on its isolating host bacterial strain was set to 100%.

### Treatment of the K5-2 phage with methyl-methane sulfonate (MMS)

The K5-2 phage was treated with 0.33% MMS for 45 or 75 min at 37 °C. To stop the reaction, an equal volume of cold 40% sodium thiosulfate was added, and this mixture was incubated at 37 °C for 10 min and then cooled on ice. Briefly, we followed a selection/amplification method to enrich for those phages that can replicate on K5 but not K69 hosts^45^. Phages were treated with MMS, and mutated phages were amplified on a K5 strain, filtered to remove bacterial debris, and then used to infect a logarithmically growing K69 strain for 5 min. This mixture was rapidly filtered before phage burst could occur. Phages able to grow on the K69 strain would attach to the cells and be eliminated from the filtrate. We then reamplified the sample on the K5 strain and repeated the cycle until titers of the filtrate were 100-fold higher on the K5 strain than on the K69 strain. This strongly selects for phage that can replicate on K5 hosts but not K69 hosts. Several plaques were picked and purified by multiple rounds of single-plaque isolation.

### Phage genomic DNA preparation and sequencing

Phage genomic DNA was extracted using a Qiagen Lamda kit (Qiagen, Alameda, CA, USA). After the phages were precipitated and lysed, phage DNA was extracted using phenol/chloroform and then precipitated with ethanol. Genomic sequencing was performed using high-throughput sequencing by the High-throughput Genome Analysis Core at the Yang-Ming Genome Research Center using the Illumina/Solexa GAII sequencing platform, with the processing and assembly methods described below. Fifty nanograms of DNA was used to construct a sequencing library by using an Illuminacompatible Nextera DNA sample preparation kit (Epicentre), according to the manufacturer’s instructions. The constructed library was quantified with quantitative PCR, and the library size was determined with a 2100. Bioanalyzer (Agilent) with a high-sensitivity DNA chip. Sequencing was performed by paired-end sequencing, with a 100-bp read length, with a HiSeq 2000 sequencing system (Illumina). The sequencing reads were trimmed for quality lower than Q20 and adapters, followed by *de novo* assembling with CLC Genomics Workbench (CLC bio, Aarhus, Denmark). Putative open reading frames (ORFs) were further predicted by Vector NTI and annotated by NCBI protein BLAST.

### Capsule depolymerase expression and purification

Polymersae chain reaction fragments of the phage gene encoding putative capsule polysaccharide depolymerases were cloned into a pET-28c expression vector (Novagen, Madison, WI, USA) via the EcoRI site. The resulting pET28c recombinant plasmid was transformed into an *E*. *coli* BL21 (DE3) strain. The recombinant His-tagged protein was expressed under 0.1 mM isopropyl β-D-1-thiogalactopyranoside (IPTG) induction at 16 °C overnight, followed by purification from the soluble fraction using Ni-NTA (Qiagen) according to the manufacturer’s instructions and SDS-PAGE analysis.

### Alcian blue staining

The exopolysaccharide extracts (containing both capsular polysaccharide (CPS) and liposaccharide) were purified by a modified hot water–phenol extraction method, as described previously^23, 65^. Briefly, 1 mL of bacteria cultured overnight in LB were harvested and resuspended in 150 μL of water. An equal volume of hot phenol (pH 6.6; Amresco) was added, and the mixture was vortexed vigorously. The mixture was then incubated at 65 °C for 20 min, followed by chloroform extraction and centrifugation. The extracted material was incubated for overnight at 37 °C, with or without purified enzyme, and then CPS was detected with Alcian blue, as described previously^66, 67^. In brief, after electrophoresis, the gel was washed three times (5 min, 10 min, and 15 min; at 50 °C for each step) with fix/wash solution (25% ethanol, 10% acetic acid in water). The gel then was soaked (15 min in the dark at 50 °C) in 0.125% Alcian blue dissolved in fix/wash solution, and finally destained (overnight at room temperature) with fix/wash solution. CPS was visualized as blue-stained material.

### Phage adsorption experiments

We made phage adsorption experiments using a previously described method^48^. Briefly, the K5 reference strain was used as a host and determined its titer on K5 and Co39 (K30) strains. The multiplicity of infection (MOI) was 1 phage particle to 100. A sample of the phage was then incubated with the K5 reference strain for 5 min, which is long enough for the phage to attach and possibly inject its DNA, but not long enough for production of new phage particles. The mixture was then rapidly filtered. Phage particles that had attached to the cells would be eliminated from the filtrate. The filtrate was then titered on both the K5 and Co39 (K30) strains. The converse experiment was performed with the Co39 (K30) strain. As controls, similar experiments using the K8 reference strain for the incubation were performed.

### Construction of a capsule mutant of the K5 strain

The sequence of the capsular polysaccharide biosynthesis gene cluster (accession no. AB289646) of the NTUH-K9534 strain was completed^9^. The K9534 strain in which *wza* and *wzb* were mutated was constructed using a previously described unmarked deletion method^63^, which employs electroporation and selection with a temperature-sensitive vector (pKO3-Km) containing flanking regions for each target gene.

### Microscopic detection of capsules

The capsules were stained using a modified Maneval’s method^68^. Briefly, bacteria were gently emulsified in a drop of 1% Congo red on a clean microscope slide and air dried. Acid alcohol was added and used as a mordant. The slide was then flooded with crystal violet and left to stand for 3 min before rinsing gently with tap water. The slide was then blot dried using clean filter paper and viewed using an oil immersion objective (×1000 magnification).

### Serum killing assays

The survival of exponential-phase bacteria in nonimmune human serum was measured as previously described^65^. Briefly, a log-phase inoculum of 2.5 × 10^4^ colony forming units (CFUs) was mixed at a 1:3 vol/vol ratio with mixed nonimmune human serum donated by 5 healthy volunteers. The final mixture, comprising 75% nonimmune serum by volume was incubated at 37 °C. For time-course studies, the bacteria/serum mixture, comprising 75% nonimmune serum by volume, was incubated at 37 °C for 1, 2 and 3 hours. The colony count was determined by plating of serial dilutions on LB agar, and the mean survival ratio was plotted. A mean survival ratio ≥1 corresponds to serum resistance.

### Phage killing assays

The bacteria (1 × 10^3^ CFUs) were incubated with phage (1 × 10^6^ PFUs, MOI = 10^3^) or (1 × 10^8^ PFUs, MOI = 10^5^) at 37 °C for 30 minutes. Then bacterial counts were determined by plating after serial dilutions and the survival ratio was calculated.

### *In vivo K. pneumoniae* infection and phage treatment

Five-week-old female BALB/cByl mice were inoculated intraperitoneally (IP) with 1.7 × 10^6^ CFUs of the *K*. *pneumoniae* NTUH-K9534. After 4 h following NTUH-K9534 infection, the mice were either inoculated intraperitoneally with 1.5 × 10^9^ PFUs of the K5-4 phage or with SM buffer only (n = 10 mice/group). Survival over a 28-day period was analyzed by Kaplan–Meier analysis with a log–rank test; differences were considered statistically significant at *P* < *0*.*05*.

### Nucleotide sequence accession numbers

The complete genome sequences of the *Klebsiella* phages K5-2 and K5-4 were deposited into GenBank under accession no. KY389315 and KY389316, respectively.

## Electronic supplementary material

Supplementary
