# Supplementary material for: Two T7-like Bacteriophages, K5-2 and K5-4, Each Encodes Two Capsule Depolymerases: Isolation and Functional Characterization
Source: Sci Rep. 2017 Jul 4;7:4624. doi: 10.1038/s41598-017-04644-2 (PMC5496888; doi:10.1038/s41598-017-04644-2)
Supplement: Supplementary file 1 — Supplementary [file 41598_2017_4644_MOESM1_ESM.pdf]

Two T7-like Bacteriophages, K5-2 and K5-4, Each Encodes Two Capsule Depolymerases: Isolation and Functional Characterization

Pei-Fang Hsieh[1], Hsiao-Hsuan Lin[1,2], Tzu-Lung Lin[1], Yi-Yin Chen[3] and Jin-Town Wang[1,4 *]

Table S1. Bacterial strains, plasmids and primers used in this study

Bacterial strain, plasmid or primer	Genotype, sequence and / or relevant description	Reference or source
**Bacteria**		
Klebsiella pneumoniae strains		
Klebsiella reference strain (42 strains)	77 K-antigen *Klebsiella* reference strains (Reference K1-K72, K74 and K79-82)	Statens Serum Institute
N1	capsule type N1 *K. pneumoniae* strain	[21]
N2	capsule type N2 *K. pneumoniae* strain	[22]
NTUH-K44	O1:K5, clinical isolate PLA strain	[63,65]
NTUH-K9534	O1:K5, clinical isolate PLA strain	[63,65]
Canada 0413, 0522, 0523 and 0525	clinical isolate bacteremia strains and were provided by Dr. Yoav Keynan	This study
Co39, Co45, Co94, DM3 and DM23	intestinal colonization *K. pneumoniae* strains	[64]
K9534 Δ*wza web*	K9534 isogenic mutant with deletion of *wza wzb* genes	This study
Escherichia coli strains		
DH10B	F^- *mcrA Δ(mrr-hsdRMS-mcrBC) Φ80* δλαχZ ΔM15 Δ*lacX74 endA1 recA1 deoR (ara leu)7697 araΔ139 galU galK nupG rpsL* λ^-	Invitrogen
BL21 (DE3)	F^- *ompT hsdSB(rB$^-$ mB$^-$) gal dcm* (DE3)	Novagen

**Plasmids**			
pGEM-T Easy	T-A cloning vector, Ap^R		Promega
pKO3-Km	pKO3 derived plasmid, with an insertion of Km resistance cassette from pUC4K into AccI site		48
pET28c-K5-2 ORF37	pET28c derived plasmid, with the K30/K69 depolymerase gene from K5-2 phage		This study
pET28c-K5-4 ORF37	pET28c derived plasmid, with the K8 depolymerase gene from K5-4 phage		This study
pET28c-K5-4 ORF38	pET28c derived plasmid, with the K5 depolymerase gene from K5-4 phage		This study
**Primers**			
K5-wzi-1F	GATGCAGCCAGACCAATGACC	K9534 *wza wzb* mutant construct	This study
K5-wzc-1R	TCCGGCATTGCTGAAACTCG		
KO5-wza-R	AGTCGCTACTAGCTTGAAGC		
KO5-wzb-F	AATCAGGAATTAGGTTATGTCATCATCAGC		
K5-wza-1R	GATCCCATACCGTGACCATC		
KO5-wzc-F	ATGTCATCATCAGCTAACAAAACG		
K8-cps-F	CTAGTCGGTTTTCTTAGAGGGG	CPS-PCR genotyping	This study
K8-cps-R	CTGTATAGTACCAGAGATAGG		
K30-69-CPS-F	CCGTTGATTTAACAATGGAAGCTC		
K30-CPS-R	GAACACATCTTTTGCAGCCAATCG		
K69-CPS-R	GCAACGCTTTTTTTGTAATAAC		
K5-GF	CCTATCTGAGAATAACGATC		
K5-GR	CTCCAATTCCGAGTTTTATCTG		
A44-2 ORF3 F	CATGTTAGACAAACTGAATCAGCCG	K5 depolymerase expression construct	This study
A44-2 ORF3 R	GGACGGAACCGCTCCGCCAG		

A44-2 ORF2 F<br>A44-2 ORF2 R	AATGGACCAAGACATTAAAAC<br>GTAGTTTTACTTTCAGGTTACCG	K8 depolymerase expression construct	This study
A44-2 ORF2 F<br>K5-2 ORF28 R	AATGGACCAAGACATTAAAAC<br>GGAACGTAGAAAGTGCAACCC	K30/K69 depolymerase expression construct	This study
enz1-F<br>enz1-R	CTGAGCGTGACTACATGACTGG<br>GCAATGGGAGCCGCCTTGATAAC	T7-like common primers	This study

S^r, resistance; Ap, Ampicillin; Km, Kanamycin

Figure S1.

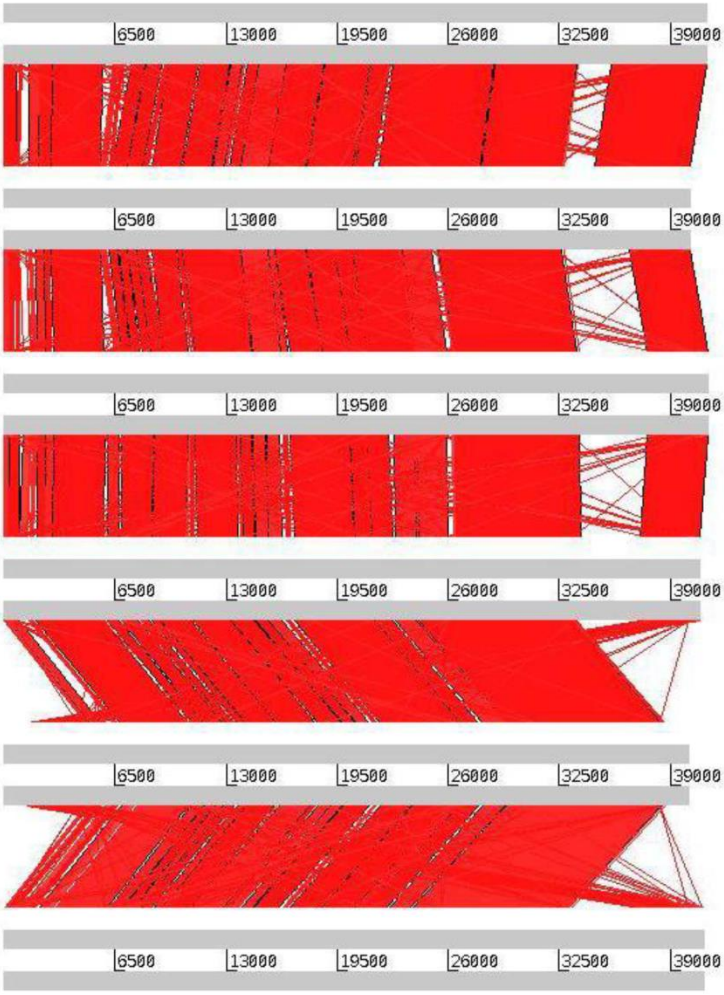
Klebsiella phage K5-2
Klebsiella phage K5-4
Klebsiella phage K11
Klebsiella phage vB_KpnP_KpV763
Klebsiella phage vB_Kp1
Enterobacteria phage K30
6500
13000
19500
26000
32500
39000

Figure S2.

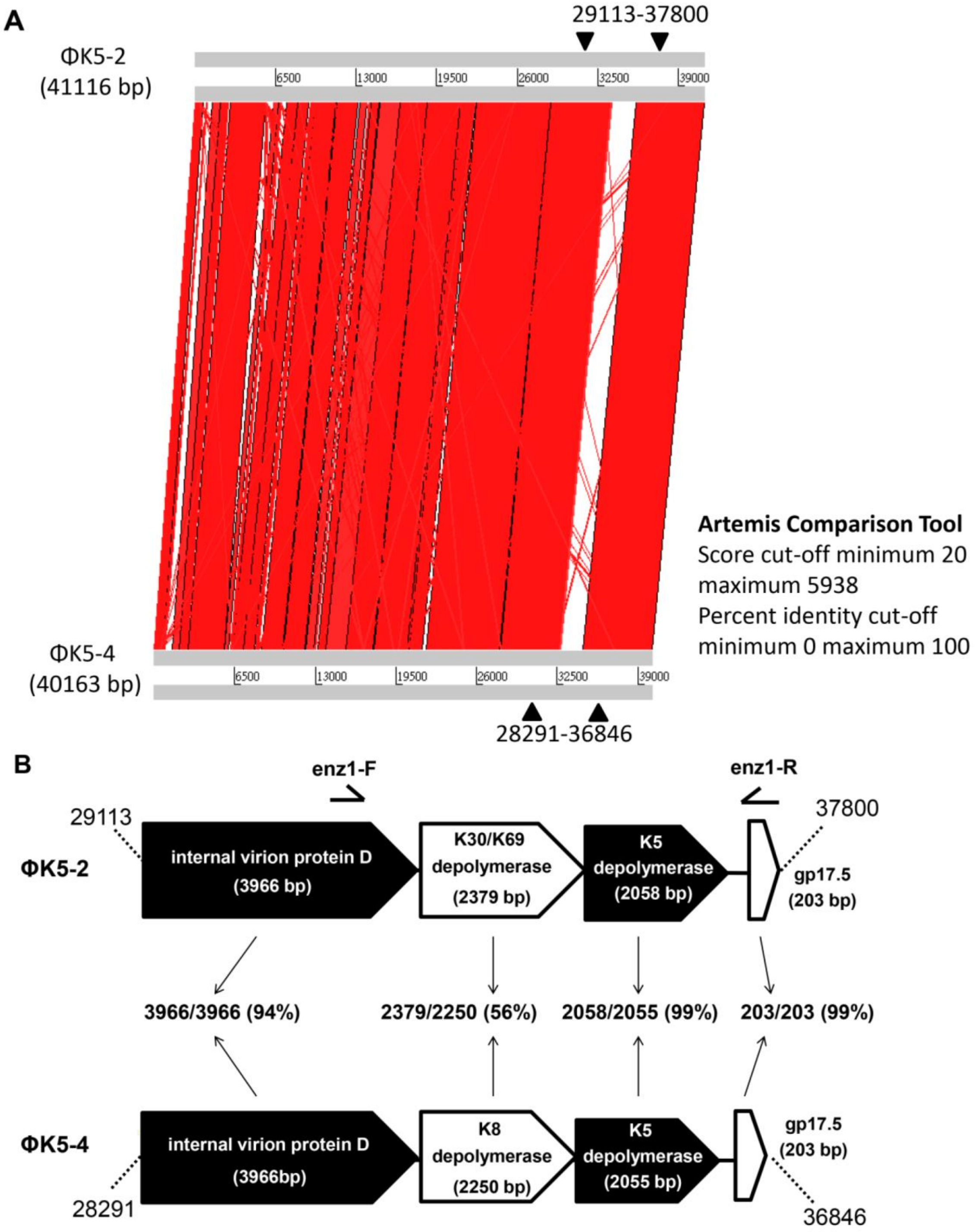
A
29113-37800
ΦK5-2
(41116 bp)
6500
13000
19500
26000
32500
39000
Artemis Comparison Tool
Score cut-off minimum 20
maximum 5938
Percent identity cut-off
minimum 0 maximum 100
ΦK5-4
(40163 bp)
28291-36846
B
enz1-F
enz1-R
29113
37800
ΦK5-2
internal virion protein D
(3966 bp)
K30/K69
depolymerase
(2379 bp)
K5
depolymerase
(2058 bp)
gp17.5
(203 bp)
3966/3966 (94%)
2379/2250 (56%)
2058/2055 (99%)
203/203 (99%)
ΦK5-4
internal virion protein D
(3966bp)
K8
depolymerase
(2250 bp)
K5
depolymerase
(2055 bp)
gp17.5
(203 bp)
28291
36846

Figure S3.

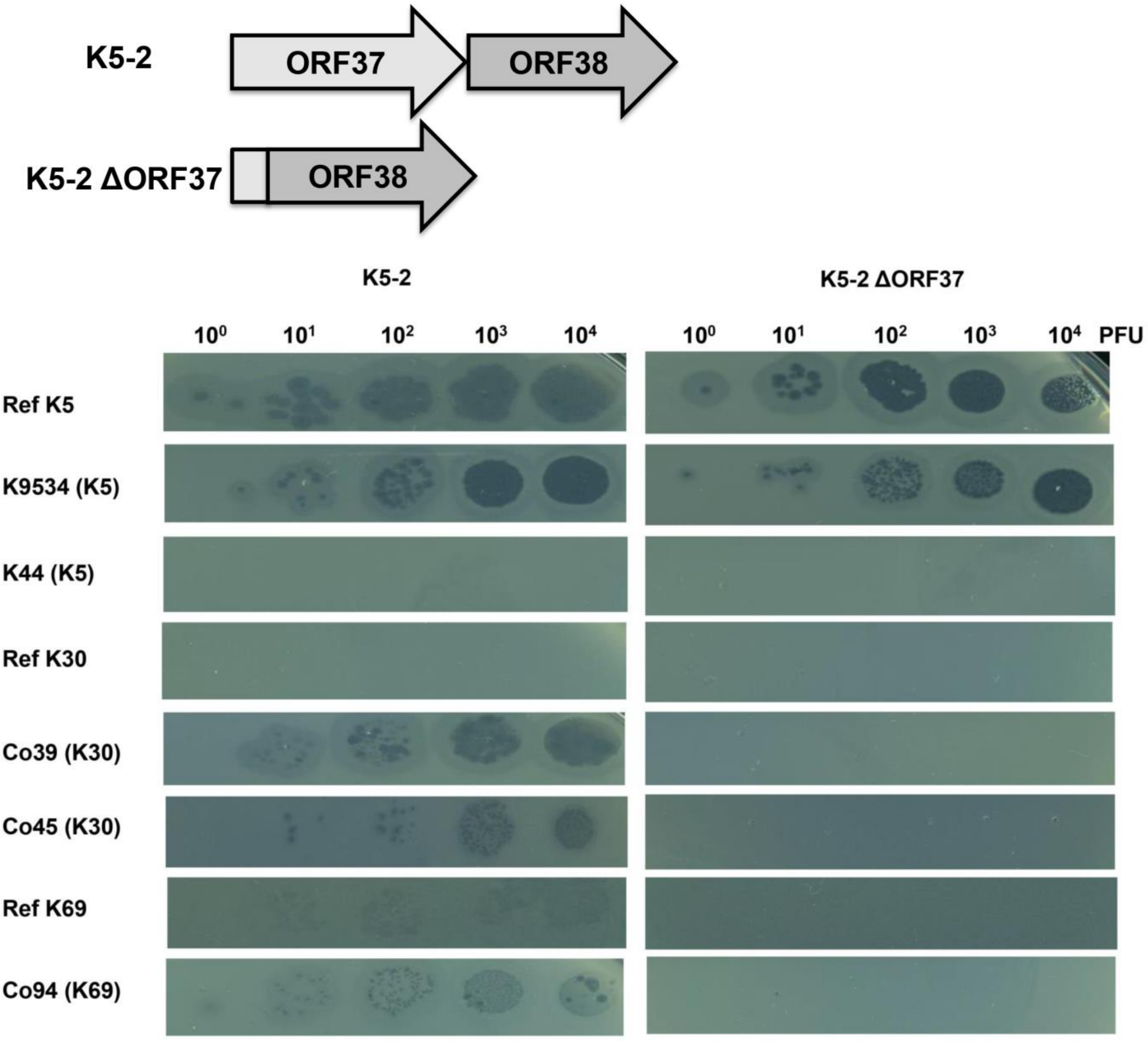
K5-2
ORF37
ORF38
K5-2 ΔORF37
ORF38
K5-2
K5-2 ΔORF37
10⁰ 10¹ 10² 10³ 10⁴
10⁰ 10¹ 10² 10³ 10⁴ PFU
Ref K5
K9534 (K5)
K44 (K5)
Ref K30
Co39 (K30)
Co45 (K30)
Ref K69
Co94 (K69)

Figure S4.

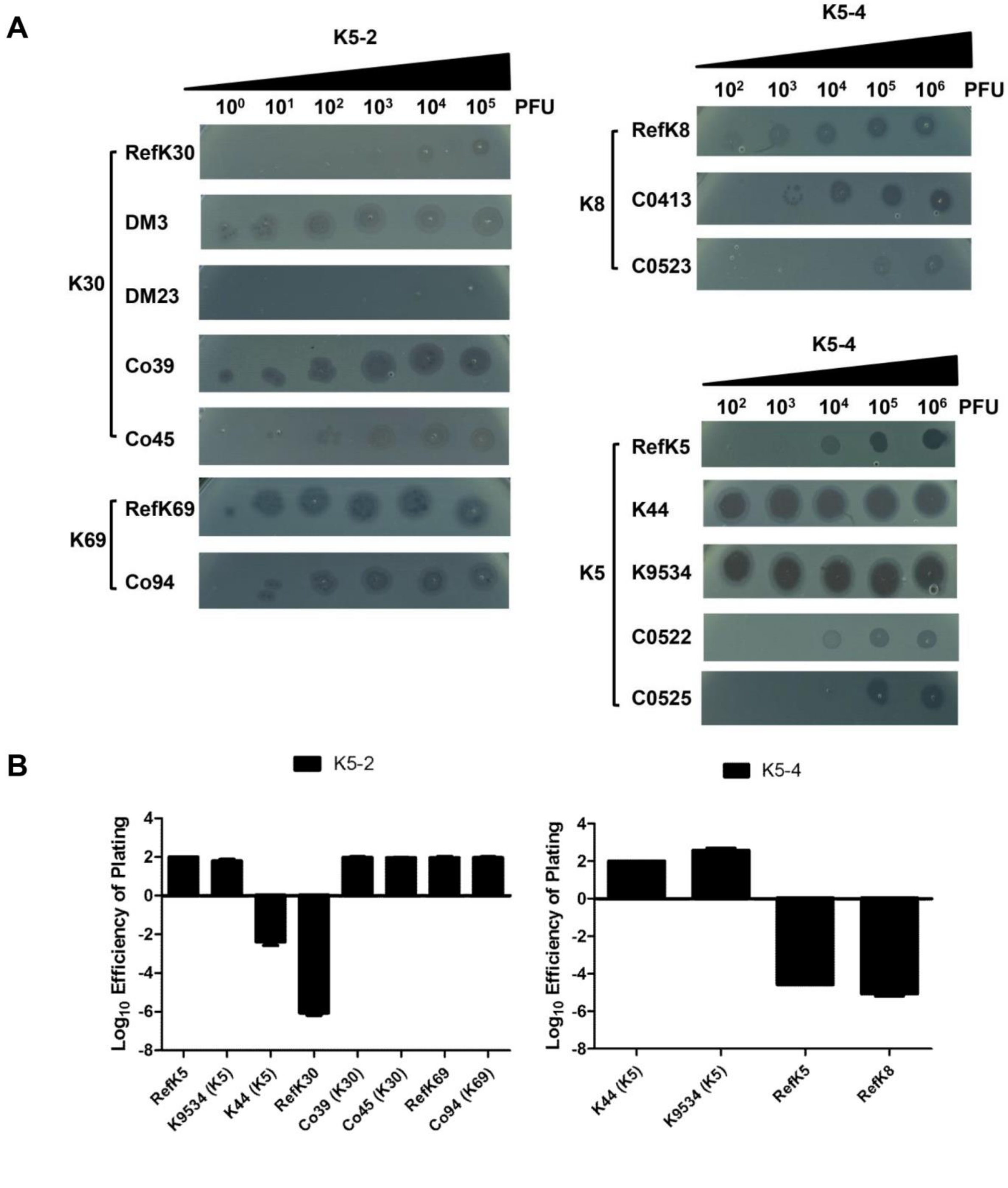
A
K5-2
10^0 10^1 10^2 10^3 10^4 10^5 PFU
RefK30
DM3
DM23
Co39
Co45
K30
RefK69
Co94
K69
K5-4
10^2 10^3 10^4 10^5 10^6 PFU
RefK8
C0413
C0523
K8
K5-4
10^2 10^3 10^4 10^5 10^6 PFU
RefK5
K44
K9534
C0522
C0525
K5
B
K5-2
K5-4
Log_{10} Efficiency of Plating
4
2
0
-2
-4
-6
-8
RefK5
K9534 (K5)
K44 (K5)
RefK30
Co39 (K30)
Co45 (K30)
RefK69
Co94 (K69)
K44 (K5)
K9534 (K5)
RefK5
RefK8

Figure Legends

Figure S1. Genome comparative analysis of five *Klebsiella* phages K5-2, K5-4, K11, vB_KpnP_KpV763, vB_Kp1 and *Enterobacteria* phage K30. Genome comparative analysis was performed using an Artemis comparison tool (score cutoffs: minimum, 20).

Figure S2. Genome comparative analysis of phages K5-2 and K5-4. ***A***, Genome comparative analysis was performed using an Artemis comparison tool (score cutoffs: minimum, 20, and maximum, 5,938; percent identity cutoffs: minimum, 0, and maximum, 100). Arrowheads indicate the regions in which 2 putative capsule depolymerases were located. *B*, Comparison of the adjacent nucleotide sequences of putative capsule depolymerases of phage K5-2 and K5-4.

Figure S3. Infectivity of phages K5-2 and its ORF37 deletion mutant among several capsular types in K5, K30 and K69 *Klebsiella* strains.

Upper panel showing schematic diagram representing the arrangement of the capsule depolymerase regions in the wild-type K5-2 and the K5-2 ORF37 deletion mutant phages. Lower panel showing differential infectivity of phage K5-2 and the K5-2 ORF37 deletion mutant on capsular types K30, K69 and K5 *Klebsiella* strains are observed.

Figure S4. Infectivity of phages K5-2 and K5-4.

A, Infectivity of phages K5-2 and K5-4 among different capsular types in the K30, K69, K8 and K5 *Klebsiella* strains. Five K30 strains (ref K30, DM3, DM23, Co39 and Co45) and two K69 strains (ref K69 and Co94) were spotted with phage K5-2 ($1\text{-}10^5$ PFUs) and K5-4 ($10^2\text{-}10^6$ PFUs), respectively. ***B***, Different infectivity of phages K5-2 and K5-4 on these tested strains are observed. Infectivity of phages K5-2 and K5-4 was represented by the efficiency of plating (log10) from three independent experiments (mean ± standard deviation [SD]).
